# Visual-spatial dimension integration in digital pathology education enhances anatomical pathology learning

**DOI:** 10.1186/s12909-022-03545-x

**Published:** 2022-07-30

**Authors:** Ken Lee Wan, Arkendu Sen, Lakshmi Selvaratnam, Mohd Imran Mohd Naing, Joon Joon Khoo, Pathmanathan Rajadurai

**Affiliations:** Jeffrey Cheah School of Medicine and Health Sciences, Monash University Malaysia, Jalan Lagoon Selatan, 47500 Bandar Sunway, Selangor Malaysia

**Keywords:** Anatomical pathology, Histopathology, Pathology education, Digital pathology, Three-dimensional (3D) imaging, Technology-enhanced learning, Technology in medical education, Medical interns

## Abstract

Literature review demonstrated a surprising lack of publications on digital e-learning pathology resources for senior medical undergraduates and interns. An interactive Digital Pathology Repository (iDPR) integrating two- and three-dimensional (2D, 3D) high-resolution anatomical pathology images with correlated digital histopathology was developed. The novel iDPR was rigorously evaluated using mixed methods to assess pathology knowledge gains (pre- and post-tests), quality impact analysis (questionnaire), user feedback (focus group discussions) and user visual behaviour (eye gaze tracking analysis of 2D/ 3D images).

Exposure to iDPR appeared to improve user pathology knowledge, as observed by significantly increased test scores on topic-related quizzes (*n* = 69, *p* < 0.001). In addition, most users were highly satisfied with the key design elements of the iDPR tool. Focus group discussion revealed the iDPR was regarded as a relevant online learning resource, although some minor technical issues were also noted. Interestingly, visual behaviour trends indicated that specific diagnostic pathological lesions could be correctly identified faster in 3D images, when compared to 2D images.

The iDPR offers promise and potential in pathology education for senior clinical students and interns, gauging from both qualitative and quantitative positive user feedback. With incorporation of image annotations and interactive functionality, and with further technology development, this would prove a useful tool for diagnostic pathology and telepathology. As images with added visual-spatial dimension can provide enhanced detail and aid more rapid diagnosis, future applications of the iDPR could include virtual reality or holographic images of anatomical pathology specimens.

**Supplementary Information:**

The online version contains supplementary material available at 10.1186/s12909-022-03545-x.

## Introduction

Irrespective of medical specialty or general practice, competency as a practising doctor is undeniably dependent on a firm foundation in pathology. Whilst recognized as a core subject encompassing the aetiology, behaviour and effects of human disease, pathology is taught primarily in the early pre-clinical years of most medical schools [1, 2], whether in a traditional or integrated curriculum format. It is well recognised that a sound knowledge of pathology underpins a host of routine clinical skills including the ordering and interpretation of tests for patients, confirmation of diagnoses based on disease pathogenesis, patient safety and even infection control [3]. Upon graduation, interns or Pre-Registration House Officers (PRHOs) have limited access to traditional pathology learning resources such as gross pathology specimens, histopathology slides or pathology museums and this comes paradoxically at a phase of their training when they are confronted daily with disease management. Furthermore, pathology attachments are usually excluded from the customary set of internship rotations (comprising the 4 basic specialties of internal medicine, general surgery, obstetrics and gynaecology, and paediatrics) whilst clinical pathology education at non-teaching hospitals has been reported to be minimal [4, 5]. In a study on preparedness of PRHOs exposed to a new integrated curriculum in the United Kingdom, junior medical doctors alarmingly reported a ‘lack of knowledge’ in pathology and therapeutics [6]. Such a ‘pathology education gap’ in the late clinical or pre-intern years has been similarly highlighted in several other medical courses, even persisting into internship [2, 7–10]. As such, this clinical pathology hiatus in medical education needs to be seriously addressed [7].

Usage of digital learning resources in medical education, especially e-learning resources, has been reported to be widely accepted amongst pre-clinical medical students studying conceptually difficult topics like leukaemia [11]. In a rare randomised controlled study, students were reportedly more receptive to educational adventure-games (game-based e-learning) and achieved better test scores compared to a cohort using traditional paper-based materials, thus, reinforcing the importance of e-learning as a valuable self-directed learning tool [12]. An online survey of digital gross pathology developed from photographs of surgical resection specimens prior to formalin fixation received encouraging evaluations from laboratory staff, with perceived room for improvement in terms of their diagnostic accuracy [13]. However, this application lacked correlation with associated histopathology images. Autopsy reports incorporating annotated gross and microscopic slide images have obtained largely positive feedback from users [14].

The incorporation of 3D technologies to enhance learner experience and performance has offered many advantages of traditional gross teaching such as ability to rotate and orient the portable specimen without the restrictions imposed by biohazardous specimens [15, 16]. Owing to the highly accurate colour and texture reproducibility through high-quality printing, the common or rare pathology specimen is not only durable, but can be accessed anywhere and anytime outside the pathology grossing room [15, 16]. Through dynamic and immersive three-dimensional (3D) visualisation, learning the anatomy of complex organs such as the brain, was easier to follow and reduced instruction time when compared with viewing static 2D images [17, 18]. In addition, a 3D-augmented curriculum has demonstrated improvement in long-term retention of gross anatomy information and proven beneficial in supplementing anatomy education in veterinary medicine [19]. A Canadian study demonstrated that when Virtual Reality (VR) is used in the 3D mode, it provides equivalent test results as seen in traditional laboratory examinations [20].

## Study goals

In this paper, the research paradigms of cognitive load theory and the cognitive theory of multimedia learning are used to allow more effective instructional visualisation [21]. Using the same research paradigm [21], the research goals of the effectiveness of 3D visuospatial instructional content in a skill-learning scenario can be addressed.

Despite increased implementation of e-learning in medical schools, systematic literature review [22, 23] identifies a serious ‘pathology education gap’, i.e., a lack of published data on digital learning resources on pathology (both anatomical pathology and histopathology) targeted appropriately for clinical students and medical interns. Hence, a readily accessible, e-learning tool with interactive 3D images of gross pathology may offer an innovative solution to improve exposure to clinical pathology at these critical phases in medical training. In order to address this pathology education gap, this study aims to investigate such a new digital pathology tool—an interactive Digital Pathology Repository (iDPR)—designed to integrate 2D and 3D images of anatomical pathological specimens with correlated histopathology tissue section images and evaluate the user learning and visual behaviour experiences amongst clinical students and fresh medical graduates.

The research questions for this study are as follows:

i) Can an iDPR tool improve clinical pathology learning to effectively address the reported ‘pathology gap’ in clinical education and internship?
ii) Is gaze behaviour and recognition of key identifying features enhanced when viewing 3D vs 2D pathology images?

The hypotheses of this study are as follows:

i) A digital pathology repository integrating annotated 2D and 3D images of gross anatomical specimens and histopathology slides may provide a useful online tool to bridge the ‘pathology gap’ in clinical education.
ii) Visualisation of dynamic 3D images provides a superior/enhanced learning experience compared to static 2D images of pathological specimens.

The study was based on the Design-Based Research (DBR) model (the cyclical process of the study: (Re)define problem(s), plan action, implement, evaluate and specifying findings) [24], and was divided into three phases:

i) Upon identification of a ‘pathology gap’, the framework of the iDPR website was **designed**.
ii) Subsequently, the iDPR website was **developed** to address the aforementioned gap.
iii) In order to effectively implement the iDPR website in the curriculum, the iDPR website underwent vigorous **analysis and evaluation**. The findings from this study can be used to redefine the problem and forms an iterative process typical of the DBR model, aimed to ultimately develop solutions to a problem.

## Methods

### Development and Evaluation of the interactive Digital Pathology Repository (iDPR) Website

**Fig. 1:**
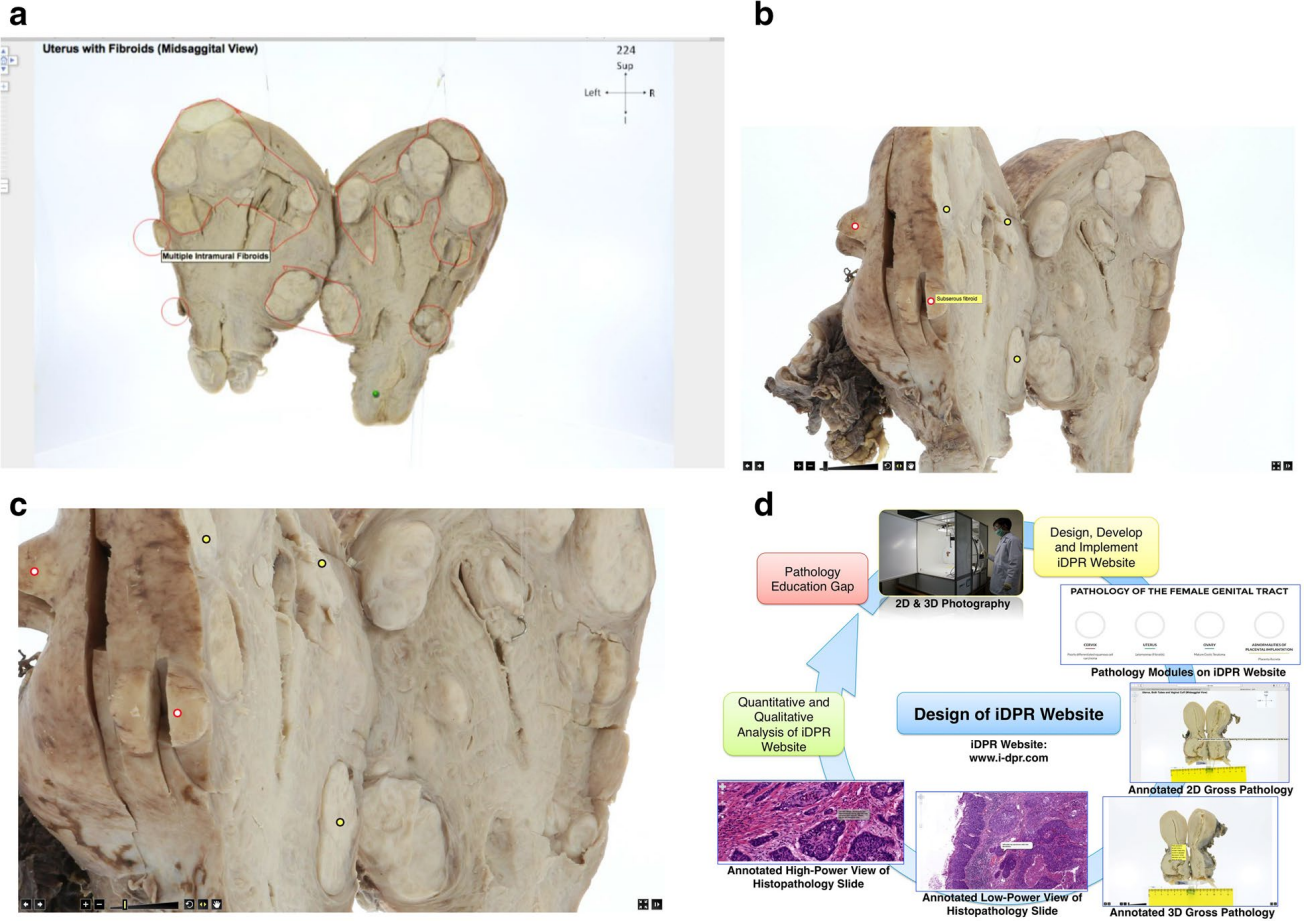
**a** Annotated 2D gross pathology image of the uterus with fibroids. **b** Annotated 3D gross pathology image of the uterus with fibroids. Users can zoom in, drag and rotate the specimen. **c** Magnified screenshot of the annotated 3D gross pathology image of the uterus with fibroids. The ultra-high-resolution is maintained even at all levels of magnification. The users can delineate each fibroid on the 3D image of the uterus. **d** Study process flow showing design, development and evaluation of the iDPR website

With regard to the Monash University’s five-year medical curriculum, pathologies related to the female genital tract are not typically covered during the third year pathology teaching of the Bachelor of Medicine/Bachelor of Surgery degree and hence there is a perceived ‘pathology gap’, specific to Monash graduates. In this pilot study, an interactive Digital Pathology Repository (or iDPR) website was designed as an e-learning tool with a focus on key female reproductive tract pathologies, topics previously identified by clinical year medical students as somewhat neglected and needing improved correlation with gynaecology postings. Specimens from the female genital tract were selected, once the reports had been finalised, and after permission for the release of tissue had been obtained from the Pathology Laboratory, Subang Jaya Medical Centre. As a learning resource, the iDPR module integrated both two- and three-dimensional images of selected gross pathology specimens (including cervical carcinoma [cervix], fibroids [uterus] and teratoma [ovary]); related histopathology slide sections; histopathology examination reports; relevant high-yield facts, fun facts and online links to an evidence-based clinical decision support system. The website design was initially beta-tested and later, implemented as an e-learning tool and evaluated for its pathology content and pedagogical value and technology enhancements. High-resolution 2D and 3D images of selected gross and microscopic pathology specimens of the female reproductive tract (see Fig. 1 (a-c)) were captured using a photo composer (*Jumbo Mode360, Poland*) prior to following a systematic process flow for the iDPR website development (see Fig. 1 (d)).

The iDPR website was designed to address the perceived ‘pathology education gap’ confronting pre-intern medical students, as identified in earlier literature reviews [2, 7–10].

**Fig. 2:**
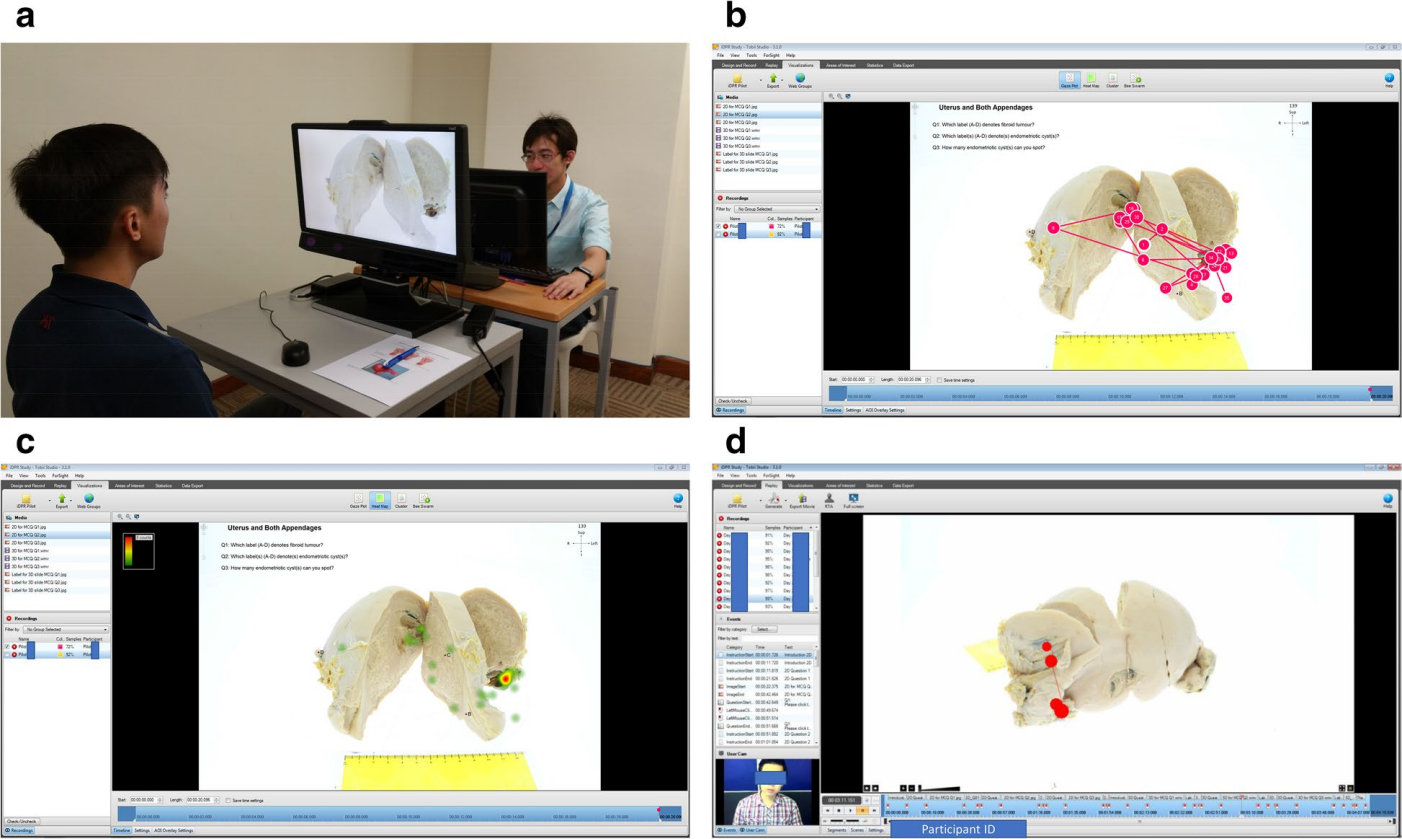
**a** Screen-based eye tracking of participant viewing image of uterus specimen. The participant observes 2D / 3D gross pathology images on the monitor with eye-tracking capability (*Tobii TX300, Sweden*), while the researcher monitors the Visualisation Data in real time. **b** Visualisation Data (Qualitative Data) recorded by *Tobii TX300, Sweden*: gaze plot—to track the participant’s gaze from the start and to the end (only for the 2D gross pathology image). **c** Visualisation Data (Qualitative Data) recorded by *Tobii TX300, Sweden*: heat map—the most frequent areas which participants focus on (only for the 2D gross pathology image). **d** Visualisation Data (Qualitative Data) recorded by *Tobii TX300, Sweden*: bee swarm—collection of gaze in a moving object (only for the 3D gross pathology image)

The 2D images of gross specimens and histopathology slides were annotated with mouse-over/ hover labels using an interactive flash builder software (*iMapBuilder HTML5, WebUnion Media Limited, Hong Kong, China*) while 3D images of gross specimens were annotated using a custom software (*360° Product Viewer: 3DRT Setup Utility v.1.3.8, Slovakia*). Both 2D and 3D images along with histopathology reports, explanatory notes including fun facts and related clinical links were subsequently uploaded onto a secure server and web domain (www.i-dpr.com) prior to beta-testing. In order to measure any gains in pathology knowledge, students were subjected to pre-/ post-tests based solely on website content. The impact quality of the iDPR website on users was evaluated qualitatively through both a questionnaire and focus group discussion. In addition, comparative eye tracking of 2D vs 3D images was measured quantitatively through visual behaviour analysis of participants using video-based eye tracking equipment (*Tobii TX300, Sweden*; see Fig. 2 (a)). The aforementioned true quantitative data was extracted from ‘Statistics’ icon (*Tobii TX300, Sweden*), while the partial qualitative data, which can be observed in real-time on the researcher’s screen (*Tobii TX300, Sweden*; see Fig. 2 (b-d)), were not analysed as they were not standardised.

The study was approved by the Monash University Human Research Ethics Committee (MUHREC) and the MBBS Ethics Committee on 10^th^ February 2016. The participants were required to voluntarily sign an informed consent form; consenting volunteers from an established medical school in Malaysia, comprised fourth year and final year medical students and also fresh medical graduates (pre-interns awaiting housemanship appointments). All methods were performed in accordance with the relevant ethics and institution guidelines and regulations.

### Pre- and post-knowledge gain test

Face validity of pre- and post-knowledge gain test was verified with academic tutors (*n* = 3) not involved in the study. The “knowledge” domains assessed in pre-tests (and identical post-tests) served to determine basic identification and comprehension of core pathologies related to the female reproductive tract (Supplementary 1).

The minimum sample size needed for acceptable saturation for this knowledge gain test was calculated at 34 participants (comparison of mean difference between two dependent pairs with 0.5 moderate effect size, generating power of 80%) using G*Power 3.1, Germany.

Consenting study participants (*n* = 69) who reviewed the iDPR website were subjected to a series of pre- and post-tests to measure the suitability of the e-learning tool for pathology learning needs and learning outcomes.

All statistical analyses were done using *IBM SPSS Statistics Version 28 Software.*

The total pre- and post-test scores were checked for skewness using One-Sample Kolmogorov–Smirnov test (*n* = 69) and if the data was skewed, comparison of the total pre- and total post-test scores (matched data) was analysed using Wilcoxon Signed Ranks test. Conversely, if the matched data were not skewed, paired t-test would be used.

### Quality impact analysis of iDPR website

Evaluation of the website was carried out through a feedback survey instrument based on a 5-point Likert Scale with options ranging from “Strongly Disagree” to “Strongly Agree”. The impact of the iDPR website (if any) was assessed for its educational content, multimedia and user interface, navigation, accessibility/technical issues, interactivity and user motivation. Although participants (*n* = 69) remained anonymous, socio-demographic data were included in this questionnaire. Respondents were also given the opportunity to include open-ended written feedback in an additional comments section. Thirty positive statements contained in the survey instrument (Supplementary 1–2) were adapted from pre-existing surveys for e-learning courses [25, 26] and the final formulated instrument used in this study demonstrated excellent internal consistency (Cronbach’s alpha 0.943).

### Focus group discussion

Ten voluntary participants were recruited to form two focus groups, comprising fourth year medical students (*n* = 5) and pre-interns/ fresh medical graduates awaiting housemanship (*n* = 5), to discuss their recent experiences with the iDPR website. The traditional size of a focus group of 10–12 people can be considered too large for education-related topics of discussion and hence, 5–8 participants are usually recommended, especially if the discussion involves expertise or strong emotions [27]. The focus group sessions (45 minutes each) were audio-recorded and later transcribed independently. Participants were identified through an anonymous alphanumeric format (e.g. P1, P2) to ensure confidentiality. The data were examined using qualitative data analysis software (*QSR NVivo 10*). The focus group discussions were based on a standardised set of questions:

- 1. Do you feel that there is a pathology gap, i.e. certain parts of pathology are not covered in the curriculum?
- 2. Have you used any e-learning tools for pathology? How effective would an interactive e-learning tool be in aiding clinical pathology learning?
- 3. Which image(s) (2-Dimensional (2D) vs 3-Dimensional (3D)) was better in terms of understanding and recognition of the lesions on the specimens?
- 4. In your opinion, how did you find the interactive Digital Pathology Repository (iDPR) website?
- 5. What were the strengths of the iDPR website?
- 6. What were the limitations of the iDPR website?
- 7. How could the iDPR website be improved?
- 8. How was your learning experience for pathology after visiting the iDPR website?
- 9. Overall, did you benefit from the iDPR website and are there any distinct advantages compared to other sources?
- 10. Any comments or suggestions?

### Visual behaviour analysis

High precision video-based eye-tracking equipment (*Tobii TX300, Sweden)*, was utilised to collect accurate gaze data with an in-built camera recording for visual behaviour analysis including fixation counts. For this part of the study, 2D and 3D images were taken of a separate gross pathology specimen (not included in iDPR pathology modules); i.e. uterus with multiple endometriotic cysts. Participants were required to ascertain the pathological diagnosis and count the total number of pathologic lesions. Calibration and refinement of the eye tracker system were carried out previously in a preliminary study (*n* = 4).

Subsequently, a separate sample cohort (*n* = 10) was voluntarily recruited comprising fresh medical graduates/ pre-house officers. Participants all read a standardised information sheet containing a summary of common pathologies of the female reproductive tract, in order to refresh and align their preliminary knowledge levels prior to undergoing eye tracking analysis. The setup, calibration and positioning of equipment, participant and researcher are illustrated in Fig. 2 (a), with each participant taking up to 5 minutes per session. The Framework of Instructions, 2D and 3D sequential images of the uterus with multiple endometriotic cysts (360° rotational video of the pathology image), three associated multiple-choice questions (MCQs) (two pathology identification-type questions (on fibroids and endometriotic cysts) and one pathology enumeration question (on counting the number of endometriotic lesions) were embedded in a PowerPoint slide timeline layout. The slideshow timing for 2D and 3D was fixed at 20 seconds each while instruction slides ran for a duration of 10 seconds each. Output measures of fixation counts were recorded as the amount of fixation (or gaze points) directed towards specific parts of an image (compared to other parts), typically indicating that more visual attention is directed there.

Quantitative data (the aforementioned sample cohort, *n* = 10) comparing number of pathologic lesions mentally counted (e.g. endometriotic cysts) when viewing 2D vs 3D images were analysed for inter-group association (Fisher’s Exact Test), while mean differences in fixation count also when viewing 2D vs 3D images were analysed via continuous variables, i.e. Independent t-test, if the differences are not skewed using One-Sample Kolmogorov–Smirnov test (*n* = 10).

## Results

### Participants and background

In total, sixty-nine participants completed the pre-test and post-test evaluations (pathology content based) and quality impact analysis of the iDPR website with the following study cohort demographics: ages ranged from 22–27 years (mean = 24.1, SD = 1.2), 60% being female and more than two-thirds of Chinese ethnic origin. Out of the 69 participants, 19 (27.5%) fourth year medical students, 12 (17.4%) final year medical students and 38 (55.1%) fresh medical graduates/ pre-house officers were involved.

### Pre- and post-knowledge gain test

After viewing the designated pathology modules on female reproductive tract pathology via the iDPR website, participant scores increased significantly in relation to content knowledge of these topics, i.e. overall median scores: pre-test 2.0 (IQR =  ± 1) vs post-test 3.0 (IQR =  ± 2) (*p* < 0.001, *n* = 69), analysed using Wilcoxon Signed Ranks test, as the participant scores were skewed, Kolmogorov–Smirnov test (*p* < 0.05, *n* = 69).

### Quality impact analysis of iDPR Website

**Fig. 3:**
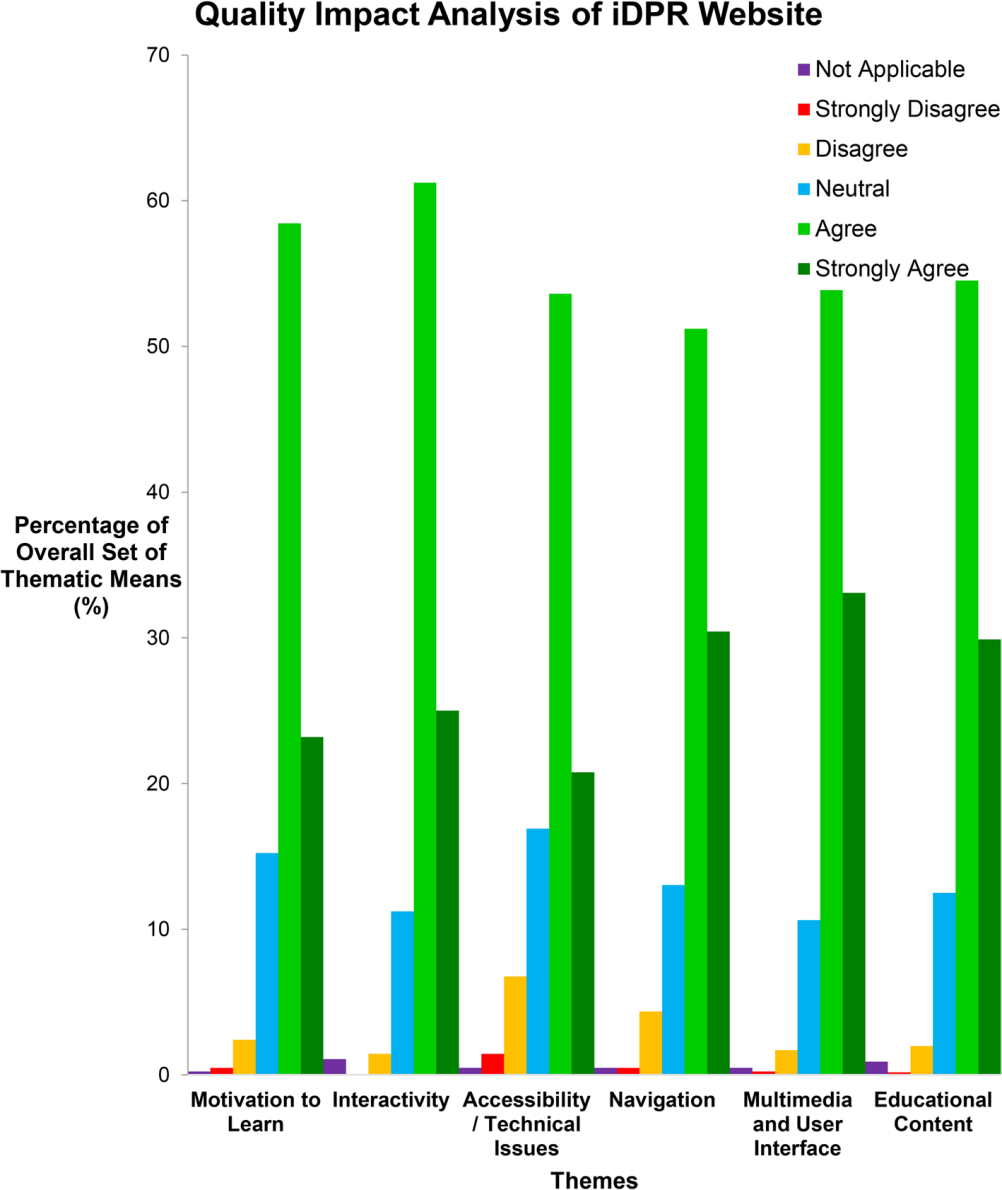
Summary evaluation of iDPR website with a focus on pedagogical, functional and technical impacts as an e-learning tool (showing mean % responses by senior medical students/ pre-interns)

Feedback evaluation of this e-learning tool was highly encouraging across the key pedagogical, functional and technical themes assessed. Interestingly, over 80% of users (*n* = 69) responded positively (strongly agreed/agreed) to the educational content, multimedia and user interface, navigation, accessibility, interactivity and motivation to learn that the iDPR website encouraged (Fig. 3) (Supplementary 2).

### Focus group discussion

Combined key findings from the two focus groups conducted were summarised according to perceived strengths, limitations and suggested areas of improvement (see Table 1). During the discussion, students from the undergraduate medical school under study readily acknowledged an apparent ‘pathology education gap’ associated with the topic of female reproductive pathology (albeit covered in brief during their pre-clinical years). One of the 10 participants defined the ideal pathology learning tool as: *“An ideal resource targeted to medical students with a goal of maximizing their learning experience should ideally include 2D and 3D images depending on subject, topic and specimen that is trying to be explained.”*

**Table 1:** Summary of combined focus group data analysis based on user experiences with iDPR website

Strengths	Limitations	Improvements
•Excellent, fantastic, useful, effective, reliable, relevant, intuitive resource •Great enhancement tool •Good tool for learning & revision •High-resolution 2D, 3D and slide scanned images •Good specimens •High-yield facts •Evidence-based •Complete information •More interesting, fun and pictures •Histopathology reports	•[web browser] •Mobile phone somewhat incompatible •Absence of graphics in high-yield facts •Longer loading time and slow Internet connectivity •Tired eyes •No audio support •[software for viewing multimedia on webpages] sometimes crashes •No physical contact with specimens	•Optimize mobile phone version •Everything on one page •Graphics in high-yield facts •More specimens of same and different pathologies / [extend to] whole body encyclopedia •Include pathophysiology •Anatomy (and [anatomical dissection] videos) and histology incorporation •Virtual tour •Running commentary •Online short video lectures •Include quizzes and answers •Include in final year exam assessment •Use [links with] free reliable websites •Interesting catchy titles

Most participants generally agreed on the many strengths of the iDPR website: *“The iDPR website is an excellent resource …, it is advanced in terms of technology and content.” “The incorporation of 2D and 3D high-resolution images and scanned images… it’s amazing.”* On the other hand, few participants expressed concerns regarding technical limitations of the iDPR website and suggested possible improvements: *“I had difficulty assessing … using [web browsers X and Y] and they didn’t work. I resorted … [mobile phone brand] surprisingly the 3D images loaded, however, the histopathology slide images didn’t work. Perhaps a support [needed] for [mobile phone brands X and Y].”*

### Visual based analysis

**Fig. 4:**
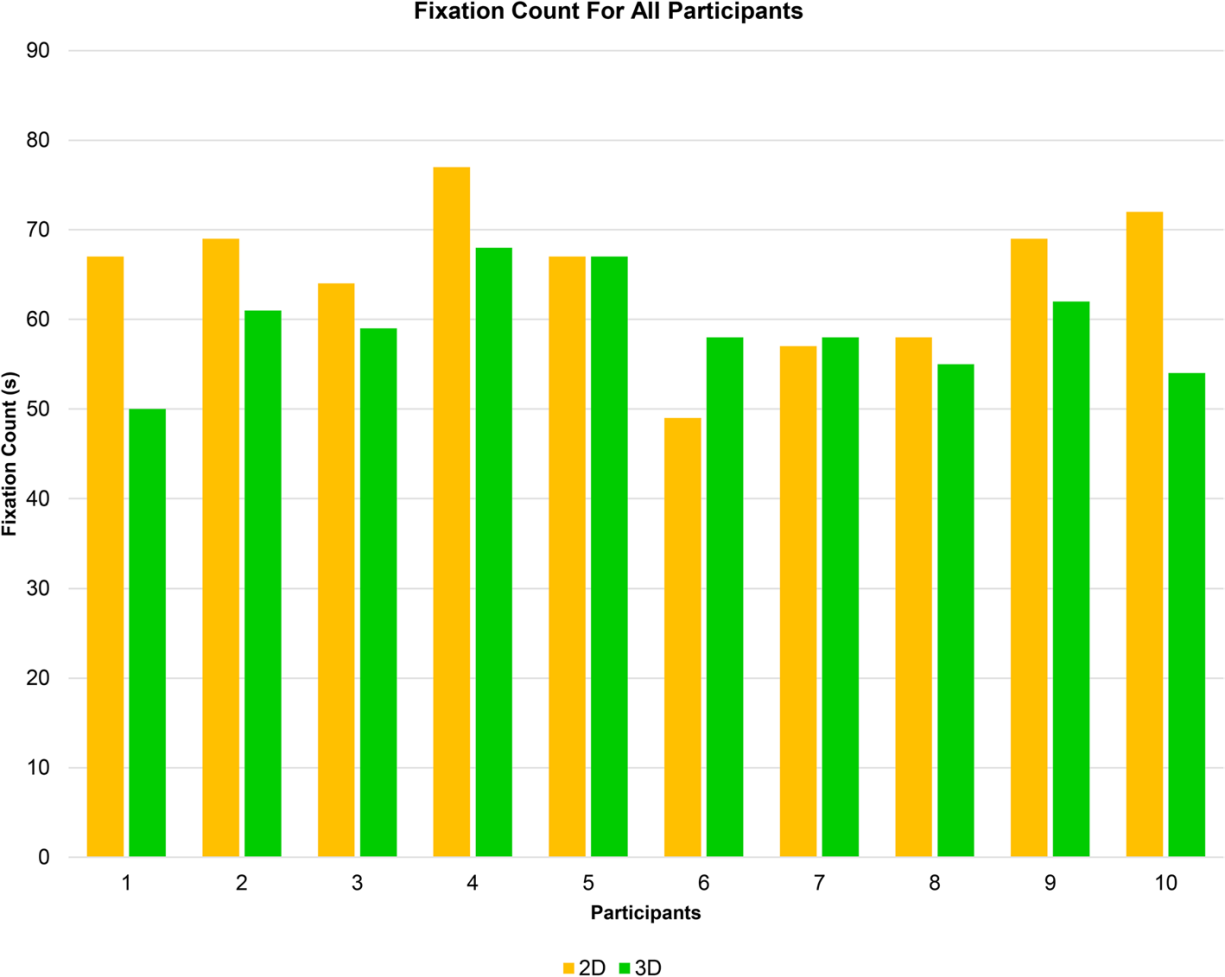
Fixation Count during observation, identification and counting the number of endometriotic lesions by participants when viewing 2D vs 3D images of the same pathology specimen. Most participants demonstrated higher fixation counts for viewing 2D compared to 3D pathology images

The visual behaviour analysis aims to compare the role of 2D and 3D gross pathology images in aiding pathology diagnostic skills. Fixation count (in seconds) is defined as the number of times the participants fixate (direct one's eyes towards) an area of interest. It is related to a cluster of gaze points which are temporally and spatially close together and are useful measures of visual attention. Out of the 10 participants involved in eye tracking analysis, 7 participants had higher fixation counts when viewing, identifying and counting endometriotic cysts from a 2D image compared to a 3D image of a pathological uterus specimen. Of the remaining 3 participants, 2 participants had less fixation counts for 3D image viewing while one participant had the same frequency of gaze points when exposed to both 2D and 3D images. Overall, although most participants demonstrated higher fixation counts for viewing 2D compared to 3D pathology images (with mean difference = 5.70 (95% CI: -8.89, 12.29). The mean difference was not skewed, (*p* > 0.05, *n* = 10) using Kolmogorov–Smirnov test and subsequently, analysed using Independent t-test (*p* > 0.05, *n* = 10), this finding is not statistically significant (Fig. 4).

## Discussion

The lack of peer reviewed publications on evaluating digital learning resources for pathology education has been previously reported, particularly highlighting the scarcity of resources designed for the needs of clinical medical undergraduates/ medical interns [22, 23]. Most maintained that this knowledge gap became more evident during their Obstetrics and Gynaecology postings during their senior clinical years. The majority of participants admitted relying solely on text or reference books to aid their clinical pathology learning. Limited supplementary learning was conducted through browser searches via the Internet (primarily Google) and through available pathology learning resources via the designated learning platform. With the aim in mind to address such a gap in the literature for pathology education, an e-learning tool for pathology—the iDPR website—was specifically conceptualised, developed and tested.

In terms of sample size for the pre- and post-knowledge gain test and quality impact analysis the minimum sample size calculated was 34, which was very well exceeded with 69 voluntary participants. Krueger has recommended 5–8 participants for expertise focus group discussions [27] which was adopted for the sample size of the two focus group discussions.

Pre-test and post-test questions to review the iDPR website were geared to the undergraduate level and house officers should also be able to answer them. Studies have shown that e-learning has helped students achieve better scores in their examination than traditional based learning [11, 12, 28, 29]. More questions could be implemented in the near future and/or incorporated in the final year assessment to properly gauge student knowledge with more specimens to be uploaded to the iDPR website. Pathology knowledge of users after viewing the iDPR website showed significant improvement, i.e. increased knowledge scores (p < 0.001). Hence, it can be concluded that the iDPR has the potential to be an effective learning tool for Pathology.

The iDPR website could be further optimised based on detailed feedback from the quantitative and qualitative analyses. Quality impact analysis of the iDPR website was largely positive with strengths mostly outweighing any reported limitations. Focus group discussions on the iDPR website were exhaustive and various factors such as high-resolution 2D and 3D gross pathology images and histopathology slides and the presence of histopathology reports were highlighted together with feedback on limitations and scope for further improvements.

The proposed application of the incorporation of 2D and 3D images of gross pathology in surgical pathology is described:- (Rare) Specimens are immortalised via 3D photography before sectioning and placed on cassettes and through wireless connection or holographic projection, the 3D photograph of the specimen with the margins can be broadcast into the Operating Theatre for the surgeon to view and decide whether or not further resection needs to be done. This technology is already used in telepathology. The presence of 3D photographic images in the histopathology report can be given to the patient in the form of a compact disk (CD). It will enable easy referral, good clinical records when the need to trace the results arises, or for any second opinions. Horn et al. have shown improvement in diagnostic accuracy with 3D imagery [13]. The digital repository of 3D gross pathology specimens will provide appropriate learning resources for anatomical pathology trainees to orientate themselves on areas to focus during their training. This approach is similar to the documented application the field of forensic pathology [30].

The novelty of the iDPR website is to visualize specimens in a manner in which they could be manipulated in their hands via 3D rendering. Even with 2D imagery, identification of subtle pathological changes compared with areas of normal tissues in gross anatomical pathology specimens remains difficult to be visualised in a non-transparent/solid organ, especially for junior medical doctors [31]. Technological advancement in digital imaging, especially computer-generated 3D visualisation, has been found to be particularly useful for tumour localisation in comparison to 2D visualisation and, more so, in surgical practice due to improved stereoscopic depth cues and orientation [31, 32]. Aner promoted learning anatomy (i.e. the study of normal body structure) in 3D as more information could be presented at once, instruction time was reduced and learning also enhanced [17]. Interestingly, Reid and Sykes emphasized that learning via 3D visualisation brings abstract concepts of Science, Technology, Engineering and Mathematics (STEM) education to life, through virtual learning [18]. For example, understanding the human brain on a static 2D image is much harder to comprehend when compared to a dynamic and immersive 3D visualisation [18].

In Iowa, Peterson and Mlynarczyk reported that 3D augmented curriculum in anatomy teaching has improved long-term retention of gross anatomy material and proven beneficial in supplementing anatomy in veterinary medicine [19]. This is an opportunity for the iDPR website to be implemented in the medical curriculum for longitudinal learning, extending from pre-clinical to clinical years and beyond including internship.

Other visual behaviour analyses have included the use of complex radiological imaging such as Positron Emission Tomography (PET) scans and functional Magnetic Resonance Imaging (fMRI) to study the brain to delineate which cortical areas are involved in 2D vs 3D perception of gross pathology images, in order to improve pathology diagnostic accuracy. Cottereau et al. have utilised MRI and high-density electroencephalogram (EEG) imaging techniques to successfully map brain areas involved in 2D motion vs 3D motion in particular V1, V4, lateral occipital complex, V3, hMT + and inferior and superior parietal complex [33]. Lee et al. combined both EEG and eye tracking device, i.e. brain computer interface to analyse differences between 2D gaze and 3D application [34]. Kober et al. have utilised only EEG to examine cortical activity when viewing 2D vs 3D VR [35], while Farahani et al. have explored the use of VR technology and a VR headset for the examination of digital pathology slides in an immersive online environment [36]. The challenges that Farahani et al. had to face included developing additional software to display the 2D whole slide images in a stereoscopic 3D virtual environment and complaints of the heavy VR headset after prolonged use [36]. Once such challenges are addressed, such 3D immersive technology could possibly usher a new era of pathology education and diagnostics.

### Limitations

In addressing the main research question of whether the iDPR e-learning tool can effectively address the reported ‘pathology gap’ in clinical education and internship, the voluntary participants have been from a private medical school university. As a result, the data may not be extrapolated to other interns from other medical schools with different clinical curricula. Based on the experience of the two co-authors, who are clinical pathologists, with vast experience with curricula contributions of many medical schools, the ‘pathology gap’ is evident. Future studies to roll out the iDPR website to other medical schools and interns and subsequently, confirming the true extent of the ‘pathology gap’.

Quality impact analysis and focus group discussion formed a major part of gathering data for quantitative and qualitative analysis of the iDPR website. The typical thematic analysis approach could also be used as a potential alternative to analyse focus group transcripts. There is an inherent limitation of self-reported or self-recalled data as it is limited by the fact that it rarely can be verified independently but accept the voluntary participants to say or write in focus groups or on questionnaires, at face value. These self-reported data contain potential sources of selective memory bias—voluntary participants have to recall their experience on the iDPR website after some time lapse.

Visual based analysis was used to study the impact of visualisation of 3D objects when compared to the traditional 2D approach. As evident from the literature review and discussion, the 2D and 3D comparisons of gross pathology were either by complex radiological imaging or EEG, or VR headset. Specifically, there was a scarcity of comparative research studies on the use of eye-tracking tools to study 3D anatomical pathology specimens. Hence, usage of eye tracking analyses in this study is an innovative approach to assess visualisation in pathology education. However, this limitation can serve as an important opportunity to explore such methods in larger research on visual impact of such 3D e-learning tools in the near future.

The limitations of the *Tobii* tool which was used for the collection of data for the visual based analysis included:

i) Incompatibility with all types of files and media.
ii) Inability to support the actual annotated 3D pathology image, the researcher compromised by using a screen recorder to record the 3D rotation of the pathology image, hence, the annotations were lost and had to be supplemented with an extra 5-s slide of 2D pathology image with labels (A-D) for answering the questions.
iii) Inability to track gaze plots (the first object the participant focuses on and subsequently when the participant stops focusing) for moving videos (3D pathology image) hence, the comparison with the 2D pathology image could not be accomplished.
iv) Inability to apply other methods of variables to the analyses, i.e. ‘time to first fixation’, to be recorded and computed as the participant will need to close their eyes before looking at the images; and ‘time for mouse clicks’. This was beyond the scope of this study according to the research questions relevant to the researcher but something to consider in different studies in the near future.

Since eye tracking is logistically complicated and costly, a limited number of participants (*n* = 10) were recruited. Even though, the results demonstrated a rather large mean difference of 5.70 when comparing the standardised 2D and 3D images of pathological uterus with endometriotic cysts, participants were mostly faster when viewing and detecting pathological lesions for 3D compared to 2D images, however, this data is not statistically significant, given the constraint of small sample size. Thus, 3D imaging may be more suitable when making a diagnosis when compared to 2D gross pathology images. Although, the eye tracking device is powerful enough to measure the fixation count for both ‘still pictures’ (2D pathology image) and ‘motion pictures’ (3D pathology image), however, the ability to track gaze plots (the first object the participant focuses on and subsequently when the participant stops focusing) can only be utilised in the former with the current paraphernalia. Thus, hindering the comparison of gaze plots between 2D image and 3D image (Fig. 2 (b-d)). This can be improved with future studies involving newer and cheaper generations of eye tracking tools and increasing sample size. This was beyond the scope of this study and is something to consider for future studies. In this study, only specific learning modules-related to the female reproductive tract pathology- were designed and developed for the iDPR website. The lack of funds and manpower made the developmental and analytical phase of the study rather small.

To make definitive conclusions on the use of 3D imagery as in the present project, future work on EEG combined with a more versatile eye tracking analysis or fMRI may be deployed for a larger group of participants to investigate the effectiveness of anatomic perception of the brain and visual gaze evaluation of 3D imagery.

## Conclusion

The literature review findings identified a scarcity of peer-reviewed publications on digital learning resources for pathology education geared towards clinical medical undergraduates/ interns. Addressing such a gap in the literature, an e-learning tool for pathology, iDPR website has been designed and developed in the present project. Pathology knowledge of users after viewing the iDPR website showed significant improvement, i.e. increased knowledge scores (*p* < 0.001). Hence, it can be concluded that the iDPR was indeed an effective learning tool for Pathology. Quality impact analysis of the iDPR website quantitatively was largely positive with strengths mostly outweighing the reported limitations. Focus group discussions on the iDPR website were exhaustive and various strengths such as high-resolution 2D and 3D gross pathology images and histopathology slides and the presence of histopathology reports together with feedback on limitations and scope for further improvements. Although not statistically significant, the time taken to view and detect pathological lesions was mostly less for 3D compared to 2D images. Hence, 3D may be better at making a diagnosis when compared to 2D gross pathology images. To make definitive conclusions on the use of 3D imagery as in the present project, future work on EEG combined with a more versatile eye tracking analysis or fMRI may be deployed for a larger group of participants to investigate the effectiveness of anatomic perception of the brain and visual gaze evaluation of 3D imagery.

The iDPR website has promising potentials in pathology education for both undergraduate and postgraduate students’ cohorts; clinical pathology for house officers/interns; and diagnostic pathology or telepathology for specialists/consultants. In the future, the iDPR website can be further revolutionised by incorporating Augmented Reality (AR) tools, VR environment or even holographic images of gross pathology specimens to view such 3D morphology of the gross pathology along with histopathology slides.

## Supplementary Information

**Additional file 1.** Pre- and Post-Knowledge Gain Test and Quality Impact Analysis for the iDPR Website.

**Additional file 2.** Participant responses to Quality Impact Analysis Survey Questionnaire for iDPR Website According to Themes & Sub-Themes (Quantitative Study).

## Data Availability

The datasets used and/or analysed during the current study are available from the corresponding author upon reasonable request.

## References

[1] Dorland WAN (2009). Dorland's pocket medical dictionary.

[2] Magid MS, Cambor CL (2012). The integration of pathology into the clinical years of undergraduate medical education: a survey and review of the literature. Hum Pathol.

[3] Munson L, Craig LE, Miller MA, Kock ND, Simpson RM, Wellman ML (2010). Elements of Good Training in Anatomic Pathology. Veterinary Pathol Online.

[4] Ford J, Pambrun C (2015). Exit competencies in pathology and laboratory medicine for graduating medical students: the Canadian approach. Hum Pathol.

[5] Wakefield D (2007). The future of medical museums: threatened but not extinct.

[6] Lempp H, Cochrane M, Seabrook M, Rees J (2004). Impact of educational preparation on medical students in transition from final year to PRHO year: a qualitative evaluation of final-year training following the introduction of a new Year 5 curriculum in a London medical school. Med Teach.

[7] Naritoku WYMDP, Vasovic LMD, Steinberg JJMD, Prystowsky MBMDP, Powell SZMD (2014). Anatomic and Clinical Pathology Boot Camps: Filling Pathology-Specific Gaps in Undergraduate Medical Education. Arch Pathol Lab Med.

[8] Hsieh CMMD, Nolan NJMD (2015). Confidence, Knowledge, and Skills at the Beginning of Residency: A Survey of Pathology Residents. Am J Clin Pathol.

[9] Yorke RF (2000). Informed evaluation of pathology residency programs: A guide for pathology resident candidates. Arch Pathol Lab Med.

[10] Hung T, Jarvis-Selinger S, Ford JC (2011). Residency choices by graduating medical students: why not pathology?. Hum Pathol.

[11] Morgulis Y, Kumar RK, Lindeman R, Velan GM (2012). Impact on learning of an e-learning module on leukaemia: a randomised controlled trial. BMC Med Educ.

[12] Boeker M, Andel P, Vach W, Frankenschmidt A (2013). Game-Based E-Learning Is More Effective than a Conventional Instructional Method: A Randomized Controlled Trial with Third-Year Medical Students. PLoS ONE.

[13] Horn CL, DeKoning L, Klonowski P, Naugler C (2014). Current usage and future trends in gross digital photography in Canada. BMC Med Educ.

[14] Pritt B, Gibson P, Cooper K, Hardin N (2004). What Is a Picture Worth?: Digital Imaging Applications in Autopsy Reports. Arch Pathol Lab Med.

[15] Bois MC, Morris JM, Boland JM, Larson NL, Scharrer EF, Aubry MC, Maleszewski JJ (2021). Three-Dimensional Surface Imaging and Printing in Anatomic Pathology. J Pathol Inform.

[16] McMenamin PG, Hussey D, Chin D, Alam W, Quayle MR, Coupland SE, Adams JW (2021). The reproduction of human pathology specimens using three-dimensional (3D) printing technology for teaching purposes. Med Teach.

[17] Aner Y. Thinking of learning 3D anatomy? Think again. Kenhub. 2016. Available from: https://www.kenhub.com/en/library/learning-strategies/thinking-of-learning-3d-anatomy-think-again. Accessed 01 Feb 2021.

[18] Reid B, Sykes W. Learning in 3D: Making STEM Real. Advanc-ed.org. 2016. Available from: http://www.advanc-ed.org/source/learning-3d-making-stem-real. Accessed 30 May 2016.

[19] Peterson DC, Mlynarczyk GS. Analysis of traditional versus three‐dimensional augmented curriculum on anatomical learning outcome measures. Anat Sci Educ. 2016;9(6):529–36. 10.1002/ase.1612 27078503

[20] Brewer-Deluce D, Bak AB, Simms AJ, Sinha S, Mitchell JP, Shin D, Saraco AN, Wainman BC (2021). Virtual Reality Bell-Ringer: The Development and Testing of a Stereoscopic Application for Human Gross Anatomy. Anat Sci Educ.

[21] Castro-Alonso JC, Ayres P, Sweller J, Castro-Alonso J (2019). Instructional Visualizations, Cognitive Load Theory, and Visuospatial Processing. Visuospatial Processing for Education in Health and Natural Sciences.

[22] Sen A, Selvaratnam L, Wan KL, Khoo JJ, Rajadurai PA. Virtual histopathology - essential education tools to ensure pathology competence for tomorrow’s medical interns. INTED2016 Proceedings. 2016 ISI Conference Proceedings Citation Index. ISBN: 978–84–608–5617–7.

[23] Wan KL, Sen A, Selvaratnam L, Khoo JJ, Rajadurai PA. Addressing the ‘Pathology Gap’ in Clinical Education and Internship: The Impetus to Develop Digital (3D) Anatomic Pathology Learning Resources. INTED2019 Proceedings. 2019 ISI Conference Proceedings Citation Index. ISBN: 978–84–09–08619–1.

[24] Kim P, Suh E, Song D (2015). Development of a Design-Based Learning Curriculum through Design-Based Research for a Technology-Enabled Science Classroom. Education Tech Res Dev.

[25] Zaharias P (2004). A usability evaluation method for e-learning courses.

[26] Balaban I, Bubas G, Pipan M. Key elements of an e-learning course evaluation survey: An empirical validation. Interactive Collaborative Learning (ICL), 2011 14th International Conference on 2011 Sep 21 (pp. 336–343). IEEE.

[27] Krueger RA, Casey MA (2009). Focus groups : a practical guide for applied research.

[28] Lam AKY, Veitch J, Hays R (2005). Resuscitating the teaching of anatomical pathology in undergraduate medical education: Web-based innovative clinicopathological cases. Pathol.

[29] Dick F, Leaven T, Dillman D, Torner R, Finken L (1998). Core morphological concepts of disease for second-year medical students. Hum Pathol.

[30] iGene Malaysia with GCS Agile Australia to Launch a Network of Digital Autopsy Facilities in Australia [Internet]. GCS Agile. 2016. [cited 6 June 2022]. Available from: https://www.gcsagile.com.au/news/igene-malaysia-with-gcs-agile-australia-to-launch-a-network-of-digital-autopsy-facilities-in-australia/.

[31] Jurgaitis J, Paskonis M, Pivoriunas J, Martinaityte I, Juska A, Jurgaitiene R (2008). The comparison of 2-dimensional with 3-dimensional hepatic visualisation in the clinical hepatic anatomy education. Medicina (Kaunas).

[32] Alaraimi B, El Bakbak W, Sarker S, Makkiyah S, Al-Marzouq A, Goriparthi R (2014). A Randomized Prospective Study Comparing Acquisition of Laparoscopic Skills in Three-Dimensional (3D) vs. Two-Dimensional (2D) Laparoscopy. J Int Soc Surg/Société Internationale de Chirurgie.

[33] Cottereau BR, McKee SP, Norcia AM (2014). Dynamics and cortical distribution of neural responses to 2D and 3D motion in human. J Neurophysiol.

[34] Lee EC, Woo JC, Kim JH, Whang M, Park KR (2010). A brain–computer interface method combined with eye tracking for 3D interaction. J Neurosci Methods.

[35] Kober SE, Kurzmann J, Neuper C (2012). Cortical correlate of spatial presence in 2D and 3D interactive virtual reality: An EEG study. Int J Psychophysiol.

[36] Farahani N, Post R, Duboy J, Ahmed I, Kolowitz BJ, Krinchai T (2016). Exploring virtual reality technology and the Oculus Rift for the examination of digital pathology slides. J Pathol Inform.

