# Supplementary material for: Visual-spatial dimension integration in digital pathology education enhances anatomical pathology learning
Source: BMC Med Educ. 2022 Jul 30;22:587. doi: 10.1186/s12909-022-03545-x (PMC9339176; doi:10.1186/s12909-022-03545-x)

**SUPPLEMENTARY 1: Pre- and Post-Knowledge Gain Test and Quality Impact Analysis for the iDPR Website**

*Required

Student ID*:

Age*:

Gender*:

*Mark only one oval.*

- Male
- Female

Ethnicity*:

*Mark only one oval.*

- Malay
- Chinese
- Indian
- Others:

Current Year of Study*:

*Mark only one oval.*

- 4th Year Medical Student
- Final Year Medical Student
- Fresh Medical Graduates / BMedSc (Hons) Researchers
- Interns / House Officers (First Year)
- House Officers (Second Year)
- Other:

**Pre-test Questionnaire**

Interactive Digital Pathology Repository (iDPR) website is an interactive Pathology e-learning tool using 2-Dimensional and 3-Dimensional virtual images of pathological specimens annotated with interactive labels and integration of related gross pathology and histopathology information. The iDPR serves as a learning resource to reinforce pathology self-study at individual convenience (especially in the latter clinical years of medical school and also for interns / house officers). It should also offer a similar 3D learning experience as manipulating an actual specimen but without physically visiting a pathology museum. This questionnaire is designed to obtain feedback of the impact of iDPR on your pathology learning gains – if any – and to establish its usability as an e-learning tool. All information will be treated anonymously. Thank you for taking part in this survey.

Please select the most appropriate response.

**Figure 1**


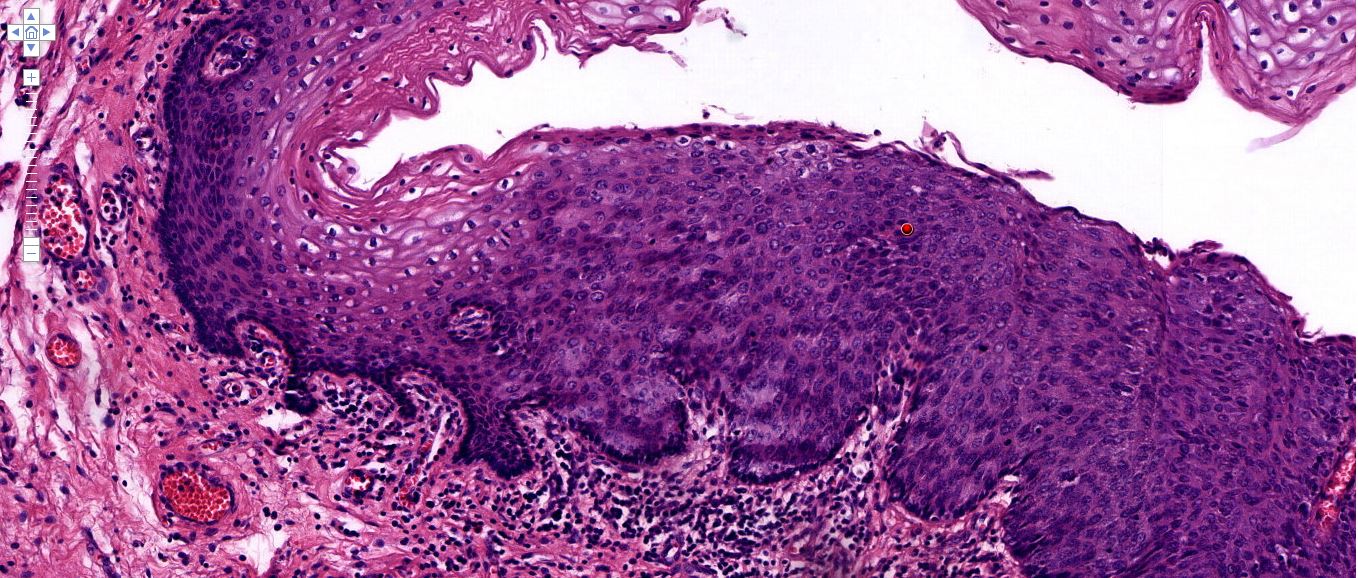


Question 1: Figure 1 shows a biopsy of the cervix. What is the grading and report based on the red label on the histopathology slide shown in Figure 1? *

Mark only one oval.

o Normal epithelium with HPV changes

o Mild squamous dysplasia (CIN 1) / Low grade squamous intraepithelial lesion

o HPV related changes (CIN 1) / Low grade squamous intraepithelial lesion

o Moderate squamous dysplasia (CIN 2) / High grade squamous intraepithelial lesion

o Severe squamous dysplasia (CIN 3) / High grade squamous intraepithelial lesion

**Figure 2**


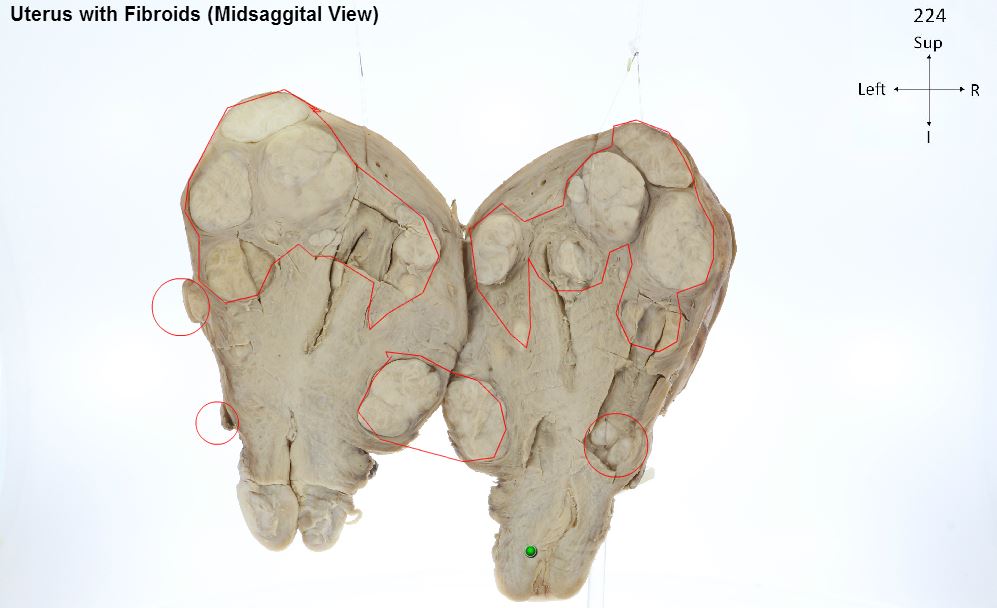


Question 2: Figure 2 shows uterus with fibroids. The fibroids are located _________. *

Mark only one oval.

o Intramural and submucosal

o Intramural and subserosal

o Intramural, submucosal and subserosal

o Submucosal, subserosal and extramural

o Intramural, submucosal, subserosal, extramural and intraluminal

**Figure 3**


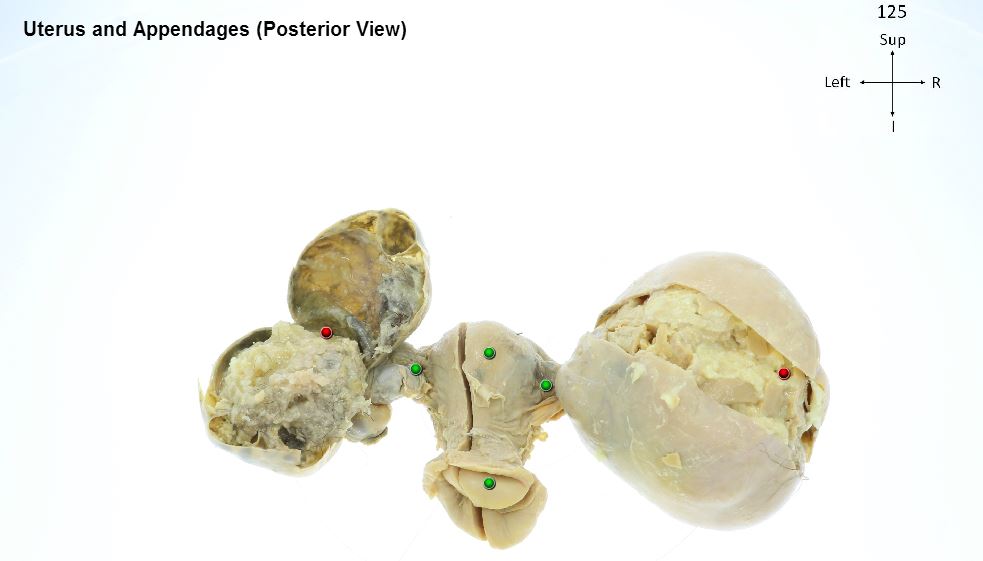


Question 3: Figure 3 shows uterus and both appendages. What is the pathological finding seen in Figure 3? *

Mark only one oval.

o Bilateral ovarian mature cystic teratoma (dermoid cyst)

o Endometriosis

o Bilateral mucinous ovarian carcinoma

o Bilateral clear cell carcinoma of the ovaries

o Polycystic ovary syndrome

Question 4: In a mature cystic teratoma, there is a predominance of ectodermal derivatives. Which of the following are derived from ectoderm? *

Mark only one oval.

o Smooth muscle, bone, teeth, cartilage, fat, gonads and dermis of skin

o Respiratory and gastrointestinal epithelium

o Thyroid and salivary gland tissue

o Keratinized epidermis, sebaceous and sweat glands, and hair follicles

**Please explore the iDPR website and please return to complete the remaining part of this form**

Kindly please use Google Chrome when exploring the iDPR website: [www.i-dpr.com](https://www.google.com/url?q=http://www.i-dpr.com&sa=D&ust=1461646587608000&usg=AFQjCNGkA5HVc26nox8Rr7Hx7fDS0SEq1g)

Passwords for each module:

1. Cervix - Poorly differentiated squamous cell carcinoma:

2. Uterus - Leiomyomas (Fibroids):

3. Ovary - Mature Cystic Teratoma:

The password for the last module 4. Abnormalities of Placental Implantation: Placenta Accreta will be given at the end of the post-test questionnaire.

Thank you and hope you enjoy exploring the iDPR website.

Kindly please return to complete the remaining part of this once you have finished the aforementioned modules.

**Post-test Questionnaire and Quality Impact Analysis for the iDPR Website**

**Please give your responses in relation to your recent experience with the iDPR website.**

|  | Please ✓ as appropriate | | | | | |
| --- | --- | --- | --- | --- | --- | --- |
|  | Strongly  Agree | Agree | Neutral | Disagree | Strongly  Disagree | Not  applicable |
| 1. The material used in this website is accurate and current to prepare you for rotation/posting in Obstetrics and Gynaecology.* |  |  |  |  |  |  |
| 1. The content is in concordance with the MBBS syllabus.* |  |  |  |  |  |  |
| 1. The content is written in a comprehensible way.* |  |  |  |  |  |  |
| 1. The content is written in an interesting way.* |  |  |  |  |  |  |
| 1. The 2D photographic images of gross pathology helped me comprehend pathology better than the traditional textbook learning.* |  |  |  |  |  |  |
| 1. The 3D photographic images of gross pathology helped me comprehend pathology better than the traditional textbook learning.* |  |  |  |  |  |  |
| 1. The scanned slide images of histopathology helped me comprehend pathology better than the traditional microscopy.* |  |  |  |  |  |  |
| 1. The links to other useful resources on the web are useful in learning pathology.* |  |  |  |  |  |  |
| 1. The 2D high resolution photographic images of gross pathology assist in my learning.* |  |  |  |  |  |  |
| 1. The 3D high resolution photographic images of gross pathology assist in my learning.* |  |  |  |  |  |  |
| 1. The high resolution scanned slide images of histopathology helped me comprehend pathology better than the traditional microscopy.* |  |  |  |  |  |  |
| 1. The layout of topics and subtopics in the iDPR websites are well structured.* |  |  |  |  |  |  |
| 1. The user interface of the iDPR website is intuitive.* |  |  |  |  |  |  |
| 1. The graphics and fonts (style, colour and saturation) for texts are legible and easy to read.* |  |  |  |  |  |  |
| 1. The participants can choose easily what parts they want to access, the order and studying pace.* |  |  |  |  |  |  |
| 1. The participants always know where they are in the iDPR website.* |  |  |  |  |  |  |
| 1. The iDPR website is more convenient than handling real specimens and slides on microscopes.* |  |  |  |  |  |  |
| 1. The iDPR website is easily accessible to a certain extent.* |  |  |  |  |  |  |
| 1. The pages and other components of the iDPR website download quickly.* |  |  |  |  |  |  |
| 1. The iDPR website is free from technical problems (hyperlink errors, programming errors, etc.).* |  |  |  |  |  |  |
| 1. The iDPR website uses high resolution images, high yield notes and evidence-based clinical decision support to gain the attention and maintain motivation of the participants.* |  |  |  |  |  |  |
| 1. The iDPR website provides access to a range of resources (web-links, evidence-based clinical decision support) appropriate to the learning context and for use in the real world.* |  |  |  |  |  |  |
| 1. The iDPR website engages participants in tasks that are closely aligned with the learning goals and objectives of the rotation/posting in Obstetrics and Gynaecology.* |  |  |  |  |  |  |
| 1. The iDPR website assists in highlighting and learning critical concepts.* |  |  |  |  |  |  |
| 1. The iDPR website incorporates novel characteristics.* |  |  |  |  |  |  |
| 1. The iDPR website stimulates further enquiry.* |  |  |  |  |  |  |
| 1. The iDPR website is enjoyable.* |  |  |  |  |  |  |
| 1. The iDPR website meets the learning needs of the participant.* |  |  |  |  |  |  |
| 1. The iDPR website provides participants opportunities to use the knowledge gained in the clinical setting.* |  |  |  |  |  |  |
| 1. Overall, taking all aspects into consideration, the iDPR website is effective in learning pathology for medical students and interns.* |  |  |  |  |  |  |

Please comment on any suggestions or improvements for the iDPR or its future websites:

**Post-test Questionnaire**

**Figure 1**


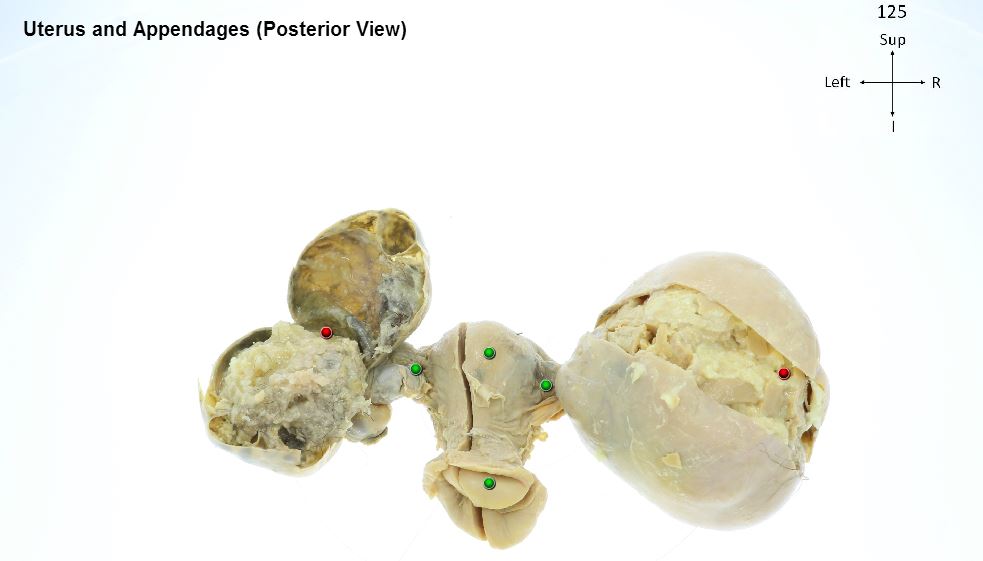


Question 1: Figure 1 shows uterus and both appendages. What is the pathological finding seen in Figure 1? *

Mark only one oval.

- - Bilateral ovarian mature cystic teratoma (dermoid cyst)
  - Endometriosis
  - Bilateral mucinous ovarian carcinoma
  - Bilateral clear cell carcinoma of the ovaries
  - Polycystic ovary syndrome

Question 2: In a mature cystic teratoma, there is a predominance of ectodermal derivatives. Which of the following are derived from ectoderm? *

Mark only one oval.

- - Smooth muscle, bone, teeth, cartilage, fat, gonads and dermis of skin
  - Respiratory and gastrointestinal epithelium
  - Thyroid and salivary gland tissue
  - Keratinized epidermis, sebaceous and sweat glands, and hair follicles

**Figure 3**


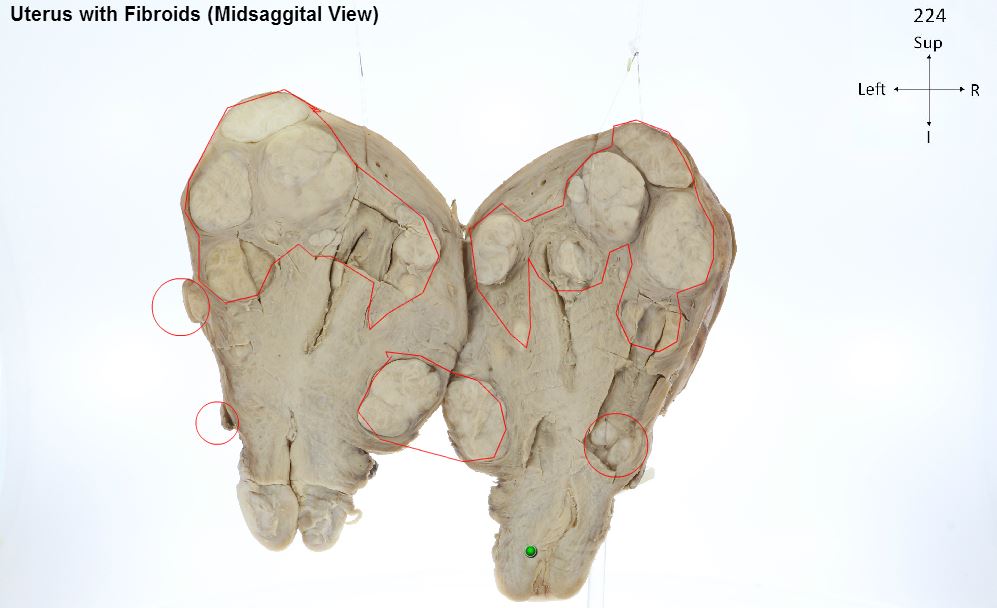


Question 3: Figure 3 shows uterus with fibroids. The fibroids are located _________. *

Mark only one oval.

- - Intramural and submucosal
  - Intramural and subserosal
  - Intramural, submucosal and subserosal
  - Submucosal, subserosal and extramural
  - Intramural, submucosal, subserosal, extramural and intraluminal

**Figure 4**


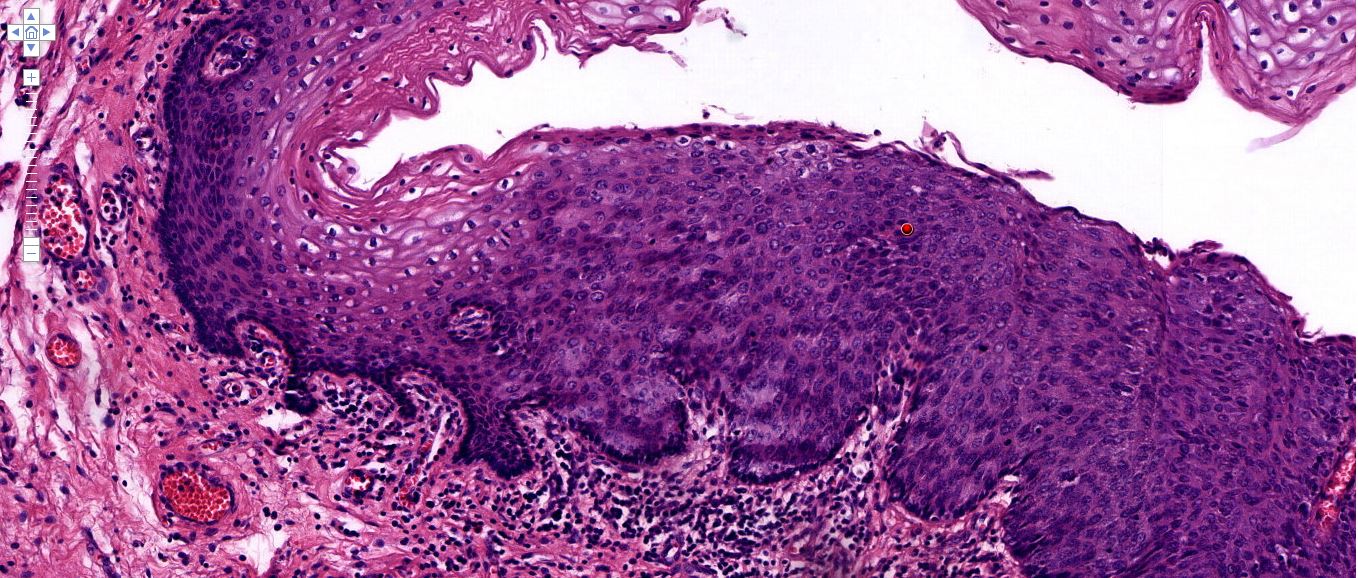


Question 4: Figure 4 shows a biopsy of the cervix. What is the grading and report based on the red label on the histopathology slide shown in Figure 4? *

Mark only one oval.

- - Normal epithelium with HPV changes
  - Mild squamous dysplasia (CIN 1) / Low grade squamous intraepithelial lesion
  - HPV related changes (CIN 1) / Low grade squamous intraepithelial lesion
  - Moderate squamous dysplasia (CIN 2) / High grade squamous intraepithelial lesion
  - Severe squamous dysplasia (CIN 3) / High grade squamous intraepithelial lesion

**Thank you for your kind participation and feedback.**

As promised, the password to the last module: 4. Abnormalities of Placental Implantation: Placenta Accreta is . Many thanks for taking your kind participation and feedback. I sincerely hope you have benefited from my humble modules on the iDPR website. I wish you all the best in your future endeavours and keep in touch. Lastly, I would to remind you of A/P Dr Khoo JJ's quote: "Pathology is the basis of medicine." Thank you.

Sincerely,

Ken Wan

Due acknowledgement are given to the following sources (Adapted from):

1. Zaharias P. A usability evaluation method for e-learning courses. Unpublished PhD Thesis, Athens University of Economics and Business, Greece. 2004.
2. Balaban I, Bubas G, Pipan M. Key elements of an e-learning course evaluation survey: An empirical validation. InInteractive Collaborative Learning (ICL), 2011 14th International Conference on 2011 Sep 21 (pp. 336-343). IEEE.
3. Questionnaire used by students to determine if they would find an educational package on posterior neck useful and, if so, what features should be included from the journal article: Pearce M, Evans D. Students developing resources for students. The Clinical Teacher, 2012, Vol.9(3), pp.178-182 [Peer Reviewed Journal].
4. Hamilton PW, Wang Y, McCullough SJ. Virtual microscopy and digital pathology in training and education. Apmis. 2012 Apr 1;120(4):305-15.
5. Appendix B (Questionnaire 2: Survey distributed to Medical Students), Payne KF, Wharrad H, Watts K. Smartphone and medical related App use among medical students and junior doctors in the United Kingdom (UK): a regional survey. BMC medical informatics and decision making. 2012 Oct 30;12(1):121.

Powered by


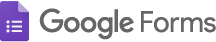

Supplement: Supplementary file 1 — Additional file 1. Pre- and Post-Knowledge Gain Test and Quality Impact Analysis for the iDPR Website. [file 12909_2022_3545_MOESM1_ESM.docx]
