# Supplementary material for: Visual-spatial dimension integration in digital pathology education enhances anatomical pathology learning
Source: BMC Med Educ. 2022 Jul 30;22:587. doi: 10.1186/s12909-022-03545-x (PMC9339176; doi:10.1186/s12909-022-03545-x)
Supplement: Supplementary file 2 — Additional file 2. Participant responses to Quality Impact Analysis Survey Questionnaire for iDPR Website According to Themes & Sub-Themes (Quantitative Study). [file 12909_2022_3545_MOESM2_ESM.docx]

**SUPPLEMENTARY 2: Participant responses to Quality Impact Analysis Survey Questionnaire for iDPR Website According to Themes & Sub-Themes (Quantitative Study)**

|  | **Frequency (%)** | | | | | | N= |
| --- | --- | --- | --- | --- | --- | --- | --- |
|  | Strongly  Agree | Agree | Neutral | Disagree | Strongly  Disagree | Not  Applicable |  |
| **Educational Content in the iDPR Website** | | | | | | | |
| 1. The material used in this website is accurate and current to prepare you for rotation/posting in Obstetrics and Gynaecology. | 20 (29.0%) | 41 (59.4%) | 5 (7.2%) | 0 | 1 (1.4%) | 2 (2.9%) | 69 |
| 1. The content is in concordance with the MBBS syllabus. | 24 (34.8%) | 38 (55.1%) | 6 (8.7%) | 0 | 0 | 1 (1.4%) | 69 |
| 1. The content is written in a comprehensible way. | 18 (26.1%) | 40 (58.0%) | 9 (13.0%) | 2 (2.9%) | 0 | 0 | 69 |
| 1. The content is written in an interesting way. | 14 (20.3%) | 34 (49.3%) | 16 (23.2%) | 5 (7.2%) | 0 | 0 | 69 |
| 1. The 2D photographic images of gross pathology helped me comprehend pathology better than the traditional textbook learning. | 20 (29.0%) | 35 (50.7%) | 13 (18.8%) | 1 (1.4%) | 0 | 0 | 69 |
| 1. The 3D photographic images of gross pathology helped me comprehend pathology better than the traditional textbook learning. | 34 (49.3%) | 30 (43.5%) | 5 (7.2%) | 0 | 0 | 0 | 69 |
| 1. The scanned slide images of histopathology helped me comprehend pathology better than the traditional microscopy. | 17 (24.6%) | 39 (56.5%) | 9 (13.0%) | 3 (4.3%) | 0 | 1 (1.4%) | 69 |
| 1. The links to other useful resources on the web are useful in learning pathology. | 18 (26.1%) | 44 (63.8%) | 6 (8.7%) | 0 | 0 | 1 (1.4%) | 69 |
| **Multimedia and User Interface of the iDPR Website** | | | | | | | |
| 1. The 2D high resolution photographic images of gross pathology assist in my learning. | 20 (29.0%) | 43 (62.3%) | 6 (8.7%) | 0 | 0 | 0 | 69 |
| 1. The 3D high resolution photographic images of gross pathology assist in my learning. | 33 (47.8%) | 32 (46.4%) | 4 (5.8%) | 0 | 0 | 0 | 69 |
| 1. The high-resolution scanned slide images of histopathology helped me comprehend pathology better than the traditional microscopy. | 22 (31.9%) | 34 (49.3%) | 10 (14.5%) | 1 (1.4%) | 0 | 2 (2.9%) | 69 |
| 1. The layout of topics and subtopics in the iDPR website are well structured. | 20 (29.0%) | 38 (55.1%) | 7 (10.1%) | 3 (4.3%) | 1 (1.4%) | 0 | 69 |
| 1. The user interface of the iDPR website is intuitive. | 20 (29.0%) | 34 (49.3%) | 13 (18.8%) | 2 (2.9%) | 0 | 0 | 69 |
| 1. The graphics and fonts (style, colour and saturation) for texts are legible and easy to read. | 22 (31.9%) | 42 (60.9%) | 4 (5.8%) | 1 (1.4%) | 0 | 0 | 69 |
| **Navigation of the iDPR Website** | | | | | | | |
| 1. The participants can choose easily what parts they want to access, the order and studying pace. | 20 (29.0%) | 44 (63.8%) | 4 (5.8%) | 1 (1.4%) | 0 | 0 | 69 |
| 1. The participants always know where they are in the iDPR website. | 13 (18.8%) | 37 (53.6%) | 15 (21.7%) | 4 (5.8%) | 0 | 0 | 69 |
| 1. The iDPR website is more convenient than handling real specimens and slides on microscopes. | 30 (43.5%) | 25 (36.2%) | 8 (11.6%) | 4 (5.8%) | 1 (1.4%) | 1 (1.4%) | 69 |
| **Accessibility/ Technical Issues of the iDPR Website** | | | | | | | |
| 1. The iDPR website is easily accessible to a certain extent. | 15 (21.7%) | 43 (62.3%) | 10 (14.5%) | 1 (1.4%) | 0 | 0 | 69 |
| 1. The pages and other components of the iDPR website download quickly. | 13 (18.8%) | 34 (49.3%) | 10 (14.5%) | 10 (14.5%) | 2 (2.9%) | 0 | 69 |
| 1. The iDPR website is free from technical problems (hyperlink errors, programming errors, etc.). | 15 (21.7%) | 34 (49.3%) | 15 (21.7%) | 3 (4.3%) | 1 (1.4%) | 1 (1.4%) | 69 |
| **Interactivity of the iDPR Website** | | | | | | | |
| 1. The iDPR website uses high resolution images, high yield notes and evidence-based clinical decision support to gain the attention and maintain motivation of the participants. | 22 (31.9%) | 37 (53.6%) | 9 (13.0%) | 0 | 0 | 1 (1.4%) | 69 |
| 1. The iDPR website provides access to a range of resources (web-links, evidence-based clinical decision support) appropriate to the learning context and for use in the real world. | 17 (24.6%) | 44 (63.8%) | 5 (7.2%) | 2 (2.9%) | 0 | 1 (1.4%) | 69 |
| 1. The iDPR website engages participants in tasks that are closely aligned with the learning goals and objectives of the rotation/posting in Obstetrics and Gynaecology. | 13 (18.8%) | 46 (66.7%) | 8 (11.6%) | 1 (1.4%) | 0 | 1 (1.4%) | 69 |
| 1. The iDPR website assists in highlighting and learning critical concepts. | 17 (24.6%) | 42 (60.9%) | 9 (13.0%) | 1 (1.4%) | 0 | 0 | 69 |
| **Motivation to Learn of the iDPR Website** | | | | | | | |
| 1. The iDPR website incorporates novel characteristics. | 14 (20.3%) | 39 (56.5%) | 13 (18.8%) | 3 (4.3%) | 0 | 0 | 69 |
| 1. The iDPR website stimulates further enquiry. | 11 (15.9%) | 40 (58.0%) | 13 (18.8%) | 3 (4.3%) | 1 (1.4%) | 1 (1.4%) | 69 |
| 1. The iDPR website is enjoyable. | 17 (24.6%) | 34 (49.3%) | 16 (23.2%) | 2 (2.9%) | 0 | 0 | 69 |
| 1. The iDPR website meets the learning needs of the participant. | 15 (21.7%) | 44 (63.8%) | 9 (13.0%) | 1 (1.4%) | 0 | 0 | 69 |
| 1. The iDPR website provides participants opportunities to use the knowledge gained in the clinical setting. | 15 (21.7%) | 45 (65.2%) | 8 (11.6%) | 1 (1.4%) | 0 | 0 | 69 |
| 1. Overall, taking all aspects into consideration, the iDPR website is effective in learning pathology for medical students and interns. | 24 (34.8%) | 40 (58.0%) | 4 (5.8%) | 0 | 1 (1.4%) | 0 | 69 |
